# Supplementary material for: Why do women attend antenatal care but give birth at home? a qualitative study in a rural Ghanaian District
Source: PLoS One. 2021 Dec 16;16(12):e0261316. doi: 10.1371/journal.pone.0261316 (PMC8675692; doi:10.1371/journal.pone.0261316)
Supplement: Attachment — Submitted filename: Response_to_reviewers_ Final_24_11_2021.docx [file pone.0261316.s002.docx]

**Response to reviewers**

We thank the editor and reviewers for evaluating our manuscript. We provide point by point responses to the comments raised by the editorial team and the reviewers. Please, find our responses to every comment/question in the table below. We have highlighted the revisions in tracked changes in the main manuscript.

PONE-D-21-15389:

| **Comments** | **Authors’ Response** |
| --- | --- |
| **Editorial Comments** | |
| 1. Please have another thorough editing for English. Please remember that the word "data" is a plural word (the singular is "datum"). Therefore, please state "data were" wherever you have stated "data is".  a) In the Background section of the Abstract, write the letter "three" with a capital T (Sustainable Development Goal Three).  b) Further, in the Results section (Traditional practices - home birth, a sign of faithfulness to husband), lines 7-8: you wrote "-- which is why she could not spontaneous vaginal delivery". Please correct this part of the sentence as "-- which is why she could not have a spontaneous vaginal delivery". | 1. We thank the review team for drawing our attention to this important error. We have now addressed and changed all the statements that indicated, “data is/was” to “data were”. These changes are marked in tracked changes in the abstract and on page 7 of the manuscript. The manuscript has also now been thoroughly edited for English by an expert.  a). We thank the review team for their kind support. We have corrected, “The Sustainable Development Goal three”, to now read “Sustainable Development Goal Three”, in the abstract, introduction, and the conclusion recommendations sections of manuscript on pages 2, 3 and 20 respectively.  b). Please, the sentence has now been corrected to read, “... which is why she could not have a spontaneous vaginal delivery”. |
| 2. In the Introduction section, first line: Please update the statistics on global number of pregnancy related deaths. According to WHO, "About 295 000 women died during and following pregnancy and childbirth in 2017". (<https://www.who.int/news-room/fact-sheets/detail/maternal-mortality>). | We are grateful to the reviewers for drawing our attention to these important updates. We have accordingly updated the statistics in the first line of the introduction as follows, “..the World Health Organization estimates that about 295 000 women died due to causes related to pregnancy and childbirth in 2017”.  Reference #1 below now replaces reference #2 also below as reference #1 in the reference list in the main manuscript.  1). World Health Organization. Maternal mortality [Internet]. 2019 [cited 2021 Nov 16]. Available from: <https://www.who.int/news-room/fact-sheets/detail/maternal-mortality>  2). World Health Organization. WHO \| Proportion of birth attended by a skilled health worker [Internet]. WHO. World Health Organization; 2008 [cited 2021 Mar 7]. Available from: <https://www.who.int/reproductivehealth/publications/maternal_perinatal_health/2008_skilled_attendants/en/> |
| 3. In the Discussion section: Please cite a reference to the paper by Paudel et al. | We thank the review team for drawing our attention to this issue. We have duly cited Paudel et al’s paper. The reference is captured as [19] on page 16 of manuscript. |
| 4. In response to Comment 7 of Reviewer#1 you have stated that "“Four of the men were husbands of the sampled women while the three were other men", but in the text "Study design, population, sampling and recruitment", lines 11-12 you have written that: "Two of the men were husbands of the sampled women who delivered at home while the five were not." Please correct this inconsistency. | Thank you for drawing our attention to this inconsistency. The response to Comment 7 of reviewer #1 was an error. The right statement is what is contained in the main manuscript on page 7. That is, “Two of the men were husbands of the sampled women while the five were other men.” |
| 5. In response Comment 14 of Reviewer#1, you have stated: "We believe the use of the word “framework” has made our argument unclear to the reviewer. We are actually referring to the analytical procedure we followed as detailed in the text. We have replaced the word “framework” with “analytical procedure". But you have still used the words "Theoretical Framework" in the text. | Thank you very much for this great input. We have now carefully read through the manuscript and replaced the word “framework” with “analytical procedure”, as marked in track changes on page 8 of the manuscript. Again, on page 4, we have replaced the sub-title “Theoretical framework of the study” with “Theoretical model of the study”, for consistency with the Andersen and Newman Behavioural theoretical model for health service utilization. |
| 6. In response to Editorial Comment 3, if possible, please upload a copy of the transcript of the interviews as a supporting document. | As we indicated in our previous response, this study is based on a dataset of 50 qualitative interview transcripts. However, we did not seek ethical permission from the participants or the ethics committee, to use data for anything else, other than the specific purposes of this study. For this reason, we do not have the explicit permission for data sharing, re-analysis or future studies. It would therefore, be inappropriate and unethical to make them available in the public domain. Furthermore, data cannot be shared publicly because the individual transcripts contain very sensitive and identifying personal information from the participants and we did not obtain consent from the participants nor the ethics committee to upload such information for public sharing. Thus, making the transcripts publicly available, will only lead to ethical violations. |
